# Diabetes Distress Among Adults With Type 1 Diabetes Mellitus in Saudi Arabia

**DOI:** 10.7759/cureus.37525

**Published:** 2023-04-13

**Authors:** Alaa A Aljohani, Esraa Y Almoghamsi, Naweed Alzaman, Mansour B Alharbi, Amjad J Bin Faidh

**Affiliations:** 1 Department of Family Medicine, Ministry of Health Holdings, Madinah, SAU; 2 Department of Family Medicine, Family Medicine Academy, Madinah, SAU; 3 Department of Internal Medicine, College of Medicine Taibah University, Madinah, SAU; 4 Department of Psychiatry, Prince Mohammad Bin Abdulaziz Hospital, Madinah, SAU

**Keywords:** saudi arabia, adults, diabetes, type 1, distress

## Abstract

Background

Psychological morbidity is clinically important for diabetes patients because it is often associated with worse glycemic outcomes. This study aimed to assess the prevalence of diabetes distress among adult type 1 diabetes mellitus (DM) patients in the Kingdom of Saudi Arabia (KSA).

Methodology

A descriptive, cross-sectional study was conducted among type 1 DM patients in KSA from 2021 to 2022. An online validated questionnaire was adopted to collect data, including demographic information, medical and social information, and Saudi Arabian Diabetes Distress Scale-17 (SADDS-17) score to assess diabetes distress.

Results

This study included 356 type 1 DM patients. Most patients were females (74%), with ages ranging between 14 and 62 years. More than half (53%) had a high level of diabetes distress with a mean score of 3.1 ± 1.23. Among those patients, the highest score (up to 60%) was related to regimen-related distress, the lowest score (around 42%) was related to diabetes-related interpersonal distress, and physician-related distress and emotional burden were reported among 55% and 51%, respectively. More than half (56%) of the patients treated with an insulin pen compared to 43% treated with an insulin pump had high diabetes distress (p = 0.049). The level of HbA1c was significantly higher among patients with high diabetic distress (7.93 ± 1.72 vs. 7.55 ± 1.65; p = 0.038).

Conclusions

Diabetes distress is prevalent among adult type 1 DM patients in KSA. Therefore, we recommend organizing a screening program for early discovery and prompt psychiatric management, incorporating diabetes education and nutrition consultation to improve their quality of life, and engaging patients in their own management to improve their glycemic control.

## Introduction

Type 1 diabetes mellitus (T1DM) is a chronic condition in which the pancreas produces little or no insulin. Patients diagnosed with T1DM need to take lifelong exogenous insulin either by multiple daily injections (MDIs) or continuous subcutaneous insulin infusion (SCII) by insulin pumps to control their blood glucose levels [1]. Epidemiological data indicate an increase of about 3-4% per year in the incidence of T1DM globally, with the age of onset younger than ever before [2].

T1DM is one of the most common disorders affecting children and adolescents worldwide and Saudi Arabia is no exception [3]. According to the Diabetes Atlas (eighth edition), in Saudi Arabia, 35,000 children and adolescents suffer from T1DM [4]; accordingly, it ranks eighth in the number of TIDM patients and the fourth country in the world in the incidence rate (33.5 per 100,000 individuals) of TIDM [3].

Diabetes distress refers to an emotional state where patients experience feelings of stress, guilt, or denial that arise from living with diabetes and the burden of self-management [5].

Psychological issues play a role in the management of any chronic illness including diabetes. These issues have a negative impact on glycemic control. Furthermore, recognizing diabetes distress is important as it is associated with worse glycemic outcomes [6].

To date, there has been limited research in Saudi Arabia on diabetes-related distress in T1DM patients. Therefore, this study aimed to estimate the prevalence of diabetes distress among T1DM patients in the Kingdom of Saudi Arabia (KSA) and identify possible associated risk factors to help guide physicians, educators, and policymakers.

## Materials and methods

Study design and area

A descriptive, cross-sectional study design was adopted. This study was conducted among T1DM patients in KSA during 2021-2022. KSA occupies about four-fifths of the Arab Peninsula, with a total population of 35,013,414 according to the General Authority for Statistics 2020 [7].

Study population, eligibility criteria, and data collection

Using patient registries, support groups, and contacts through social media, we recruited a diverse sample of adults with T1DM in KSA from central, western, eastern, northern, and southern regions. Inclusion criteria were as follows: Saudi patients aged 14 years with a diagnosis of T1DM for at least 12 months. Patients with major psychiatric/neurocognitive disorders that would inhibit their ability to participate were excluded from the study.

Sample size

A convenient non-probability sampling technique was adopted to select the required sample online. The required minimum sample size was computed using the Roasoft online sample size calculator by considering the confidence level set at 95%, the percentage picking a choice or response (50% = 0.5), and the confidence interval (0.05 = ±5). Accordingly, the required sample size was 384 patients.

Study tool

An online questionnaire was adopted to collect data in this study. The questionnaire included demographic information such as age, gender, education level, job, marital status, living area, and family history of diabetes. In addition, other questions included medical and social information such as height, weight, smoking status, type of medications used for diabetes, last glycated hemoglobin (HbA1c) level, frequency of hypoglycemia and diabetic ketoacidosis (DKA), and a history of other chronic or psychiatric illnesses.

The Diabetes Distress Scale-17 (DDS-17) is a commonly applied tool to assess diabetes distress among diabetic patients. It comprises 17 items describing possible diabetes-related problems experienced over the last month. Each item is scored on a six-point Likert scale ranging from 1 (no distress) to 6 (serious distress). The total score was computed for every patient and divided by the total items and then categorized into the following two groups: low diabetes distress (<3) and high diabetes distress (≥3). The DDS-17 has four domains: emotional burden, physician-related distress, regimen-related distress, and diabetes-related interpersonal distress [8].

Saudi Arabian Diabetes Distress Scale-19 (SADDS-17) is a pre-validated and translated self-administered online questionnaire. It was translated using the forward-backward translation from English to Arabic at King Saud University Medical City, Riyadh, Saudi Arabia, in January 2016. This version was applied in the present study [9].

Pilot study

A pilot study was conducted in a diabetic clinic to test the questionnaire’s applicability and assess the items of the SADDS-17 questionnaire. About 30 patients were included in the pilot study. These patients were not included in the main study. Additionally, the time taken to complete the questionnaire was assessed for each participant, which helped estimate the time needed for the interview. Approximately 12 minutes were needed to fill out the questionnaire by each participant.

Data entry and analysis

Data were coded, entered, and analyzed using SSPS software, version 26 (IBM Corp., Armonk, NY, USA). Categorical data were described using frequency and percentage while quantitative continuous variables were described using arithmetic mean and standard deviation. The chi-square test was used to investigate the association between two categorical variables, and Student’s t-test was applied to test compare the mean of a continuous variable between two different groups. The result was considered statistically significant if the p-value was less than 0.05.

Professional ethics

The proposal was submitted to the local research ethical committee, the Ministry of Health in Madina, for approval. Written informed consent was obtained from all respondents. A letter was attached to each questionnaire which emphasized that the confidentiality of personal data was maintained by the researcher.

## Results

The study included 356 T1DM patients out of the targeted 384 patients (response rate = 92.7%). Table 1 presents their demographic characteristics. Most patients (73.6%) were females, with ages ranging between 14 and 62 years. About two-thirds (66.1%) had bachelor or postgraduate degrees. Students represented 44.9% of the patients. Overall, 76.4% of the participants were single, and the majority of them (93.5%) lived with their families. A considerable proportion of the participants (41.9%) lived in the western region of Saudi Arabia.

**Table 1:** Demographic characteristics of type 1 diabetes mellitus patients in the Kingdom of Saudi Arabia.

	Frequency	Percentage
Gender		
Male	94	26.4
Female	262	73.6
Age in years (range)	14–62	
Educational level		
Primary school	5	1.4
Intermediate school	22	6.2
High school	94	26.4
Bachelor degree	212	59.6
Postgraduate	23	6.5
Job		
Employed	81	22.8
Unemployed	113	31.7
Student	160	44.9
Retired	2	0.6
Marital status		
Single	272	76.4
Married	70	19.7
Divorced	10	2.8
Widowed	4	1.1
Current living status		
Alone	23	6.5
With family	333	93.5
Region of residence		
Western	149	41.9
Eastern	64	18.0
Northern	24	6.7
Southern	29	8.1
Central	90	25.3

Medical and social history

**Figure 1:**
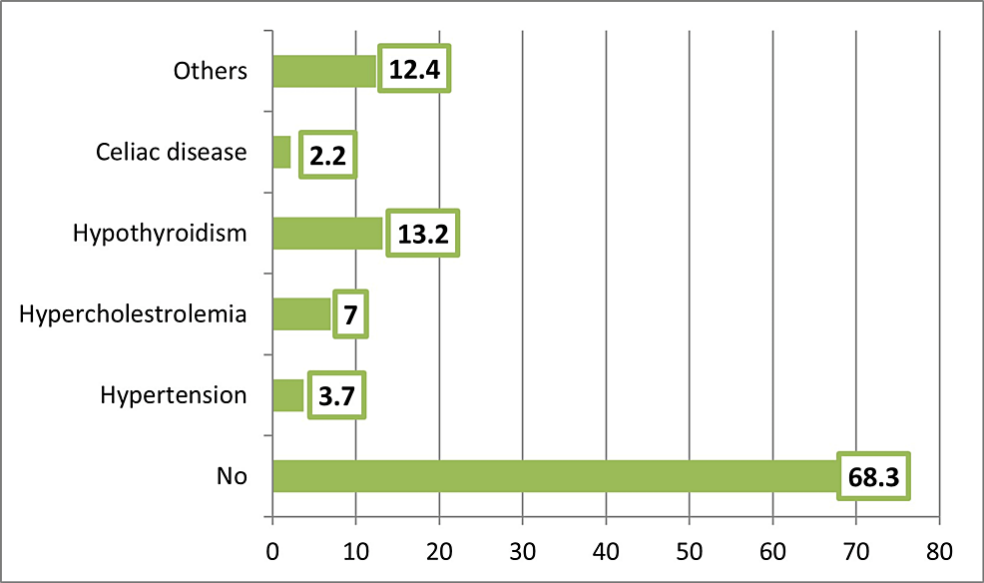
History of other chronic diseases among the participants.

Family history of diabetes was observed among almost one-third (31.2%) of T1DM patients. A history of smoking was reported by 5.9% of T1DM patients. A history of other comorbid chronic diseases was observed among 31.7% of T1DM patients, particularly hypothyroidism (13.2%) (Figure 1).

**Figure 2:**
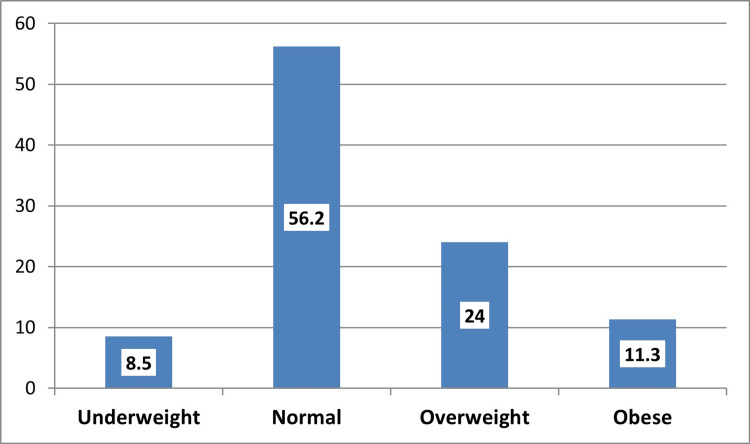
Body mass index of the participants (n = 354).

As shown in Figure 2, more than one-third of the participants were either overweight (24%) or obese (11.3%).

Duration of T1DM ranged between five and 10 years among 29% of patients whereas it exceeded 15 years among 28% of patients. The majority of patients (80%) were using an insulin pen for therapy, and almost half of the patients were using continuous glucose monitors (49%) for measuring their blood glucose. Their last HbA1c ranged between 4% and 16%, with a mean ± standard deviation of 7.75 ± 1.70. The number of hypoglycemic attacks exceeded six in the last month among 30.1% of patients, while the number of DKA attacks exceeded two among 6.7% of patients (Table 2).

**Table 2:** Diabetes-related characteristics of type 1 diabetes mellitus patients in the Kingdom of Saudi Arabia. HbA1c: glycated hemoglobin; SD: standard deviation

	Frequency	Percentage
Duration in years		
1–5	71	19.9
5–10	102	28.7
11–15	83	23.3
>15	100	28.1
Type of treatment		
Insulin pen	284	79.8
Insulin pump	72	20.2
Measurement of blood glucose		
Finger stick glucose test	180	50.6
Continuous glucose monitor	176	49.4
Last HbA1c level (n = 354)		
Range	4–16	
Mean ± SD	7.75 ± 1.70	
Number of hypoglycemic attacks in the last month		
None	25	7.0
1–3	130	36.5
4–6	94	26.4
>6	107	30.1
Number of diabetes ketoacidosis attacks in the last month		
None	255	71.7
Once	55	15.4
Twice	22	6.2
>twice	24	6.7

Diabetes distress

**Figure 3:**
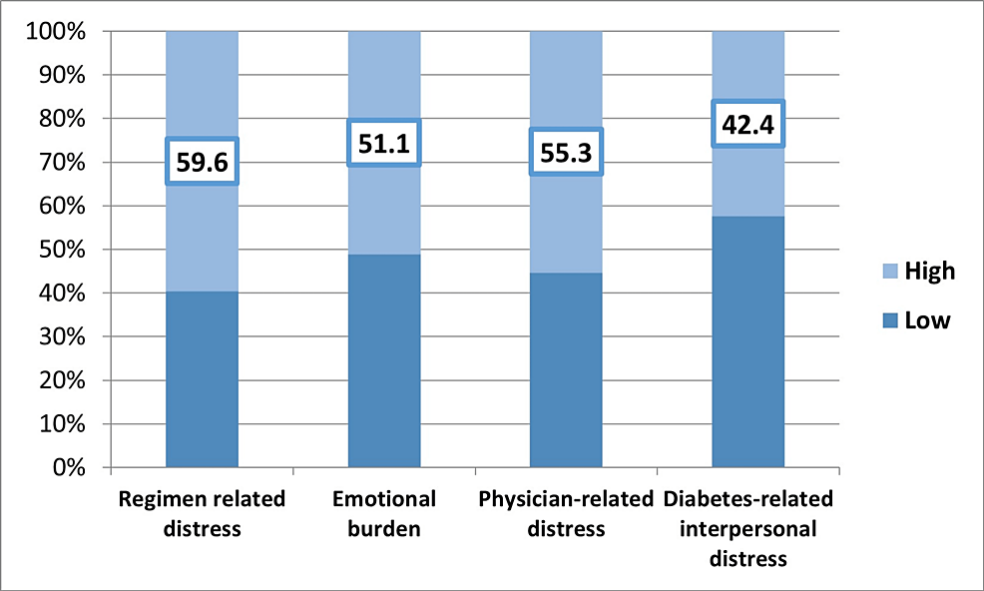
High levels of domains of diabetes distress among type 1 diabetes mellitus patients in the Kingdom of Saudi Arabia.

More than half (53.4%) of T1DM patients had a high level of diabetes distress, with a mean of 3.1 ± 1.23. The highest level of distress was regimen-related among 60% (3.25 ± 1.36), the lowest was diabetes-related interpersonal distress among 42% (2.7 ± 1.56), and physician-related and emotional burden distress was reported among 55% and 51% (3.26 ± 1.66 and 3.1 ± 1.39), respectively (Figure 3).

Factors associated with diabetes distress

Demographic Factors

None of the studied demographic characteristics of T1DM patients (gender, age, educational level, job, marital status, living status, and region of residence) was significantly associated with high level of diabetic distress (Table 3).

**Table 3:** Demographic factors associated with diabetes distress among type 1 diabetes mellitus patients in the Kingdom of Saudi Arabia. *: Chi-square test; **: Student’s t-test

	Diabetes distress		P-value
	Low	High	
	N = 166, N (%)	N = 190, N (%)	
Gender			
Male (n = 94)	46 (48.9)	48 (51.1)	0.601*
Female (n = 262)	120 (45.8)	142 (54.2)	
Age in years			
14–62, Mean ± SD	25.4 ± 8.2	25.3 ± 8.0	0.875**
Educational level			
Primary school (n = 5)	5 (100)	0 (0.0)	0.081*
Intermediate school (n = 22)	10 (45.5)	12 (54.5)	
High school (n = 94)	40 (42.6)	54 (57.4)	
Bachelor’s degree (n = 212)	97 (45.8)	115 (54.2)	
Postgraduate degree (n = 23)	14 (60.9)	9 (39.1)	
Job			
Employed (n = 81)	40 (49.4)	41 (50.6)	0.934*
Unemployed (n = 113)	53 (46.9)	60 (53.1)	
Student (n = 160)	72 (45.0)	88 (55.0)	
Retired (n = 2)	1 (50.0)	1 (50.0)	
Marital status			
Single (n = 272)	125 (46.0)	147 (54.0)	0.151*
Married (n = 70)	38 (54.3)	32 (45.7)	
Divorced (n = 10)	2 (20.0)	8 (80.0)	
Widowed (n = 4)	1 (25.0)	3 (75.0)	
Current living status			
Alone (n = 23)	10 (43.5)	13 (56.5)	0.754*
With family (n = 333)	156 (46.8)	177 (53.2)	
Region of residence			
Western (n = 149)	66 (44.3)	83 (55.7)	0.587*
Eastern (n = 64)	26 (40.6)	38 (59.4)	
Northern (n = 24)	13 (54.2)	11 (45.8)	
Southern (n = 29)	15 (51.7)	14 (48.3)	
Central (n = 90)	46 (51.1)	44 (48.9)	

Medical and Social Factors

More than half (56%) of the patients treated with an insulin pen compared to 43.1% treated with an insulin pump had high diabetes distress (p = 0.049). The HbA1c level was significantly higher among patients with high diabetic distress (7.93 ± 1.72 vs. 7.55 ± 1.65; p = 0.038). Other studied factors (family history of diabetes, smoking status, history of comorbid chronic diseases, body mass index, duration of the disease, method of measurement of blood glucose, and the number of hypoglycemic and/or DKA attacks in the last month) were not significantly associated with the level of diabetes distress (Table 4).

**Table 4:** Medical and social factors associated with diabetes distress among type 1 diabetes mellitus patients in the Kingdom of Saudi Arabia. *: Chi-square test; **: Student’s t-test

	Diabetes distress		P-value
	Low	High	
	N = 166, N (%)	N = 190, N (%)	
Family history of diabetes			
No (n = 245)	114 (46.5)	131 (53.5)	0.956*
Yes (n = 111)	52 (46.8)	59 (53.2)	
Smoking history			
No (n = 335)	158 (47.2)	177 (52.8)	0.419*
Yes (n = 21)	8 (38.1)	13 (61.9)	
History of comorbid chronic diseases			
No (n = 243)	110 (45.3)	133 (54.7)	0.450*
Yes (n = 113)	56 (49.6)	57 (50.4)	
Body mass index			
Underweight (n = 30)	14 (46.7)	16 (53.3)	0.315*
Normal (n = 199)	92 (46.2)	107 (53.8)	
Overweight (n = 85)	45 (52.9)	40 (47.1)	
Obese (n = 40)	14 (35.0)	26 (65.0)	
Duration in years			
1–5 (n = 71)	37 (52.1)	34 (47.9)	0.120
5–10 (n = 102)	42 (41.2)	60 (58.8)	
11–15 (n = 83)	33 (39.8)	50 (60.2)	
>15 (n = 100)	54 (54.0)	46 (46.0)	
Type of treatment			
Insulin pen (n = 284)	125 (44.0)	159 (56.0)	0.049*
Insulin pump (n = 72)	41 (56.9)	31 (43.1)	
Measurement of blood glucose			
Finger stick glucose test (n = 180)	76 (42.2)	104 (57.8)	0.092*
Continuous glucose monitor (n = 176)	90 (51.1)	86 (48.9)	
Last HbA1c level (n = 354)			
Mean ± SD	7.55 ± 1.65	7.93 ± 1.72	0.038**
Number of hypoglycemic attacks in the last month			
None (n = 25)	6 (24.0)	19 (76.0)	0.075*
1-3 (n = 130)	67 (51.5)	63 (48.5)	
4-6 (n = 94)	46 (48.9)	48 (51.1)	
>6 (n = 107)	47 (43.9)	60 (56.1)	
Number of diabetes ketoacidosis attacks in the last month			
None (n = 255)	122 (47.8)	133 (52.2)	0.277*
Once (n = 55)	20 (36.4)	35 (63.6)	
Twice (n = 22)	13 (59.1)	9 (40.9)	
>Twice (n = 24)	11 (45.8)	13 (54.2)	

## Discussion

T1DM can induce adverse outcomes on a patient’s physical, psychological, and social development due to the management schedule and the high rate of complications [10,11]. Moreover, diabetes distress in T1DM patients itself is associated with decreased diabetes self-care and impaired quality of life [12].

The main objectives of this study were to determine the prevalence of diabetes distress among adult T1DM patients throughout KSA, whereas other studies were conducted in a specific region or a specific population. The prevalence of diabetes distress in our study among T1DM adult patients using the SADDS-17 was 53.4% and the mean score was 3.13 ± 1.23.

A prospective cohort study was conducted among T1DM patients recruited from the Diabetes Treatment Center, Prince Sultan Military Medical City, Riyadh, Saudi Arabia, between March 2019 and October 2019. This study found a significant improvement in diabetes distress and sleep quality as well as glycemic outcomes after using FreeStyle Libre for three months in young adults with T1DM. Moreover, reductions were observed in mean DDS (3.8 vs. 2.5; p < 0.001) and Pittsburgh Sleep Quality Index (8.7 vs. 3.9; p < 0.001) scores from baseline to three months [13]. In two tertiary care centers in Jeddah, Saudi Arabia, Alyahyawi et al. assessed the impact of diabetes distress on glycemic control among adolescents and youth with T1DM. In the study, the prevalence of diabetes distress among adolescents with T1DM was 24.1%. The mean scores of Problem Areas in Diabetes and DDS were 43.56 ± 13.84 and 2.22 ± 1.05, respectively [14].

Another study conducted in the United States among adults with T1DM revealed a prevalence rate of diabetes distress of 42% [15].

Very close figures were reported from similar studies conducted in Nigeria (52%) [16], Malaysia (49%) [17], and Bangladesh (48%) [18]. However, lower figures were reported from studies conducted in South Africa (44%) [19], Brazil (31.5%) [20], Canada (39%) [21], and another previous Saudi study (25%) [22]. High rates were observed in a study conducted in Sudan (87.6%) [23]. In Iran, a rate of 63.7% was reported [24].

It is very important while interpreting these figures to keep in mind that most of these studies were conducted among type 2 diabetic patients or both types while our study targeted adults with type 1 diabetes only. In addition, differences between various studies can be attributed to the difference in healthcare systems, setting of care, demographic characteristics of patients, and health conditions, particularly HbA1c level and comorbidities [16-24].

It has been documented that type 1 diabetic patients were more likely to have higher diabetes distress than type 2 diabetic due to several factors, including their younger age at diagnosis, longer duration of the disease, having fewer coping mechanisms, higher cost of medications, more difficult therapeutic regimen, and different social, psychological, and physical stressors faced by patients [25].

In this study, high levels of regimen-related distress, physician-related distress, emotional burden, and diabetes-related interpersonal distress were reported among 60%, 55%, 51%, and 42% of T1DM patients, respectively. While in other studies from Nigeria [16], Bangladesh [18], South Africa [19], Brazil [20], and Canada [21], emotional burden and regimen-related distress had the highest score among the subscales of diabetes distress.

Studies from Nigeria [16] and Brazil [20] compared the use of insulin with oral medications and reported that distress was significant with the use of insulin in general. However, in our study, we compared the use of insulin pens versus insulin pumps and found that patients treated with insulin pens were significantly at higher risk for developing diabetes distress in comparison to those using insulin pumps. This can be attributed to the experienced pain from repeated injections, feeling inconvenienced, being hospitalized, decreasing quality of life, and fear of hypoglycemic attacks [26].

Uncontrolled diabetes, as evidenced by a higher level of HbA1c, was significantly associated with a higher level of diabetes distress in this study. The same has been documented by many studies reported from Japan [27], Jordan [28], Thailand [29], and the United States [30]. However, the relationship between poor glycemic control and its psychological changes in diabetic patients is not fully understood as it is not clear which leads to the other. Recently, Tunsuchart et al. suggested that distress had a significant impact on glycemic control and complications in patients [29]. Therefore, further longitudinal studies are warranted for a better understanding of the relationship between poor glycemic control and the psychological status of diabetic patients.

To our knowledge, this is the first national study to assess diabetes distress among T1DM patients in different Saudi Arabia regions. The strengths of the present study include the adaptation of an Arabic-language validated form of DDS among the participants. In addition, we focused our study on adults with type 1 diabetes as most similar studies were conducted on type 2 diabetic patients. However, a few limitations of this study should be mentioned, which could direct further research. First, the cross-sectional design of the study with the convenience sampling method may not represent the entire profile of diabetes distress among Saudi patients throughout the whole country. Second, the causal relationships between diabetes distress and other possible associated factors could not be established due to the cross-sectional design. Finally, other potential risk factors for diabetes distress, such as the cost of therapy and health insurance, were not investigated in this study.

## Conclusions

Diabetes distress is prevalent among adult T1DM patients in KSA. Patients treated with insulin pens and those with uncontrolled diabetes, as evidenced by a higher level of HbA1c, were more likely to develop diabetes distress than their peers. Based on these findings, we recommended organizing a screening program for adult T1DM patients for early discovery and prompt psychiatric management of diabetes distress, as well as incorporating diabetes education and nutrition consultation to improve the quality of life of such patients. In addition, particular psychiatric care should be given to insulin-treated patients and those with poor glycemic control.
